# Follow-Up of Nonarteritic Anterior Ischemic Optic Neuropathy With Optical Coherence Tomography Angiography

**DOI:** 10.1167/iovs.62.4.42

**Published:** 2021-02-26

**Authors:** Edouard Augstburger, Arnaud Ballino, Chafik Keilani, Mathieu Robin, Christophe Baudouin, Antoine Labbé

**Affiliations:** 1 Department of Ophthalmology III, Quinze-Vingts Hospital, IHU FOReSIGHT, Paris, France; 2 Quinze-Vingts Hospital, IHU FOReSIGHT, INSERM-DHOS CIC 1423, Paris, France; 3 Inserm, U968, UPMC Univ Paris 06, UMR_S968, Institut de la Vision; CNRS, UMR 7210; CHNO des Quinze-Vingts, INSERM-DHOS CIC 503, Paris, France; 4 Department of Ophthalmology, Ambroise Paré Hospital, AP-HP, University of Versailles Saint-Quentin-en-Yvelines, Versailles, France

**Keywords:** nonarteritic anterior ischemic optic neuropathy (NAION), optical coherence tomography angiography (OCT-A), microvascularization, capillary plexus

## Abstract

**Purpose:**

The purpose of this study was to describe capillary changes in patients with nonarteritic anterior ischemic optic neuropathy (NAION) using optical coherence tomography-angiography (OCT-A) and correlate the results with best corrected visual acuity (BCVA), visual field, OCT retinal nerve fiber layer (RNFL), and combined thickness of ganglion cell and inner plexiform layers (GCIPL) thicknesses.

**Methods:**

We enrolled 22 eyes with acute NAION and 30 normal control (NC) subjects in this study. Whole en face image vessel density (WiVD) was measured in the radial peripapillary capillary plexus (RPC), superficial capillary plexus (SCP), and deep vascular complex (DVC) using OCT-A. The examination was repeated at 1 (M1), 3 (M3), 6 (M6), and 9 (M9) months after presentation for NAION.

**Results:**

The initial RPC WiVD was significantly reduced in the acute NAION group compared to the NC group (*P* < 0.0001). Over the course of NAION follow-up, RPC WiVD was significantly reduced at M1 (*P* < 0.001 compared to M0) and M3 (*P* < 0.0001 compared to M1). However, there was no significant further decrease at M6 and M9. The initial SCP WiVD was significantly reduced in the NAION group compared to the NC group (*P* < 0.0001 for both). Over the course of NAION follow-up, a significant decrease was observed for SCP WiVD at M1 (*P* < 0.001 compared to M0), but no significant change was seen at M3, M6, or M9. DVC was normal in the NAION group. Correlations were found between GCIPL and SCP WiVD in the NAION acute phase (R = 0.604, *P* = 0.003) and in the M9 atrophic stage (R = 0.551, *P* = 0.009). At M9, RPC WiVD was correlated with BCVA (R = −0.562, *P* = 0.007), mean deviation (R = 0.518, *P* = 0.01), and RNFL (R = 0.655, *P* = 0.001).

**Conclusions:**

Over the course of NAION, OCT-A provided detailed visualization of retinal capillary plexus involvement.

Nonarteritic anterior ischemic optic neuropathy (NAION) is the leading cause of acute optic neuropathy in individuals over 50 years of age^1^ and results in irreversible loss of visual function.² NAION is an acute ischemia of the optic nerve head (ONH) secondary to a transient slowing of circulation in the posterior ciliary arteries (PCAs). Generally, it occurs in predisposed subjects with a small ONH, also known as a “disc at risk.” In these subjects, a triggering factor such, as an episode of prolonged nocturnal hypotension, may precipitate the development of NAION.^2^ This defect in perfusion of the ONH leads to ischemic lesions in the retinal nerve fiber layer (RNFL) and causes optic disc edema. The loss of vision is thus often discovered on awakening.^3^^,^^4^

Recently, several groups, including ours, have demonstrated a decrease in peripapillary and macular vessel density (VD) using optical coherence tomography angiography (OCT-A) in the atrophic stage of NAION.^5^^–^^7^ This decrease in VD corresponds precisely to a depletion of the functional retinal capillary meshwork. The retinal peripapillary capillaries (RPC) form an anastomotic network that runs parallel to the axons within the RNFL. Several studies^8^^–^^11^ in the past have shown that the metabolic interdependence between these structures is strong, and RPC involvement is related to the severity of the optic neuropathy. Although a decrease in peripapillary and macular capillary density was observed in the atrophic phase in our first retrospective study, it was crucial to follow patients with NAION from the acute phase to study the kinetics of this impairment. This would allow a better understanding of the mechanism. In particular, it was important to know whether the capillary damage was total at the time of diagnosis of acute NAION (and therefore probably resulting from hypoperfusion of the ONH) or whether it was progressive and therefore probably associated with edema.

In fact, the reason for this capillary damage remains partially unexplained. Due to masking artifact related to dye diffusion in the edematous stage, the microvascular peripapillary and macular changes during resorption of the edema have not yet been clearly identified in fluorescein angiography. In contrast, OCT-A does not require dye injection and has an axial and lateral resolution approaching the micron range, allowing a fine and measurable analysis of capillary networks.^12^ Disc edema has been suspected to cause a “compartment syndrome-like” phenomenon, where axonal swelling causes compression of adjacent capillaries within an increasingly crowded disc. This is the beginning of a vicious cycle in which compression of the capillaries leads to a decrease in blood flow, worsening the stasis in axoplasmic flow, and thus the axonal swelling.^3^ Indeed, most of therapeutic trials have focused on accelerating resorption of the edema, in particular through the use of corticosteroids.^13^^–^^15^ The results of these studies remain controversial,^16^ because, although corticosteroids accelerated the resorption of the edema, no improvement in acuity or visual field was demonstrated. The underlying mechanism is probably more complex, especially because the damage is not limited to the ONH but also extends into the macular area through decreased capillary perfusion and atrophy of the ganglion cell layer.^5^^,^^17^

Some recent studies have reported assessment of NAION using OCT-A in the acute phase and confirmed that capillary damage was present at the onset of the disease.^18^ In a study of 6 cases, Rebolleda et al.^19^ reported that RPC density appears to decrease during the first 3 months of resorption of the edema. Thus, the objective of the present study was to analyze by OCT-A the damage to the peripapillary and macular retinal capillaries during the acute edematous phase of NAION and during progression to the atrophic phase, and to analyze potential correlations between capillary damage and visual or OCT parameters.

## Materials and Methods

### Subjects

This prospective case-control observational study was conducted at the Quinze-Vingts National Ophthalmology Hospital in Paris, France, between January 2019 and February 2020. None of the patients included in our previous retrospective study^5^ were included in this new prospective study. All of the included patients and control subjects were informed of the data collection, and informed consent was obtained from each subject. The study was conducted in accordance with the Declaration of Helsinki and approved by our CPP-Ile-de-France Ethical Committee (number 10793). All patients referred for or diagnosed with acute NAION in the emergency department were proposed for inclusion, and then the diagnosis was confirmed by one of the investigators. The criteria for the diagnosis of NAION were: a sudden, painless unilateral decrease in visual acuity occurring in a subject over 40 years of age with optic disc edema, whether associated or not with peripapillary hemorrhage, and compatible visual field impairment, in the presence of a normal C-reactive protein blood test. All patients were also examined by the internal medicine department to screen for systemic risk factors for NAION^2^^–^^4^ (diabetes, hypertension, obstructive sleep apnea syndrome [OSAS], and dyslipidemia) and to rule out giant-cell arteritis. If in doubt, a temporal artery biopsy was performed.

An age- and sex-matched normal control (NC) group was recruited during the same period from emergency department patients or their attendants, as long as they had no impairment that could interfere with the study data: best corrected visual acuity (BCVA) > 20/20 with normal Humphrey SITA 24-2 visual field, a normal-appearing and symmetrical ONH in both eyes.

Exclusion criteria for the NAION and NC groups were refractive error > +6.00 or < −6.00 D, measured IOP > 21 mm Hg, a history of glaucoma or neurological disease, associated retinal or choroidal pathology, or a history of ocular surgery (excluding uncomplicated cataract surgery over 6 months previously).

All NAION and NC subjects underwent a full ophthalmologic examination, including an assessment of BCVA according to the standardized Early Treatment Diabetic Retinopathy Study (ETDRS) scale (BCVA was secondarily converted to a log of the minimum angle of resolution), slit-lamp anterior segment examination, applanation tonometry, fundus examination, and an automated visual field (Humphrey Visual Field Analyzer, SITA-Standard 24-2 program; Carl Zeiss Meditec, Dublin, CA, USA). Peripapillary RNFL and macular ganglion cell inner plexiform layer (GCIPL) thickness measurements were performed using the Cirrus SD-OCT (Carl Zeiss Meditec). These measurements were performed at baseline for all patients and controls and repeated at 1, 3, 6, and 9 months in the NAION group.

### OCT-A Data

All acute NAION and NC subjects were examined using OCT-A (RTVue XR100 Avanti; Optovue, Inc., Fremont, CA, USA) during the first visit. Subsequently, all measurements were repeated during the subacute (1 and 3 months after diagnosis) and chronic (6 and 9 months) phases for NAION subjects. A 4.5 × 4.5 mm scan rectangle automatically centered on the optic disc and a 6 × 6 mm scan rectangle automatically centered on the fovea were performed using the AngioVue split spectrum amplitude-decorrelation angiography algorithm. Images were obtained after pupil dilation, and images with poor quality (less than 7/10) or eye movement artifact were excluded. Automatic segmentation was used for the analysis of the various retinal vascular plexuses.

In accordance with the parameters automatically defined by the system and already published,^5^ superficial capillary plexus (SCP) was defined as the zone between 3 µm below the internal limiting membrane and 16 µm below the inner border of the inner plexiform layer (IPL). The deep vascular complex (DVC), which is a combination of the deep and intermediate capillary plexuses, was defined as the zone between 16 µm below the inner border of the IPL and 9 µm below the outer plexiform layer and outer nuclear layer junction.^20^ An eye tracker and projection artifact resolution system was included in the Optovue software. The segmentation was verified by the investigators (authors A.E. and B.A.); in cases where no reliable automatic segmentation was available, the patient was not included in the study.

VD measurements were provided automatically from the angiograms by the software. They corresponded to the percentage of surface area with blood flow over the entire selected region. For the peripapillary region, the whole en face image vessel density (WiVD) was calculated at the level of the RPC. In the macular area, the WiVD was calculated at the level of the SCP, DVC. The Optovue software included the 3D projection artifact removing algorithm,^20^^,^^21^ which was used to limit the projection of superficial macular vessels on DVC measurements.

### Statistical Analysis

All descriptive statistics were presented as the median (range). Mann-Whitney bilateral and Wilcoxon signed rank tests were used for continuous data, and a χ^2^ test for categorical data. Correlation coefficients were determined using Spearman's correlation coefficient. Values of *P* < 0.01 were considered to be statistically significant. Statistical analyses were performed with the XLSTAT 2020 software.

## Results

A total of 29 subjects with unilateral NAION and 30 NC subjects were included in this study. Among the NAION subjects, four patients were lost to follow-up and thus excluded, and three had an image quality that was considered inadequate in the initial phase and were thus also excluded. A total of 22 subjects with NAION were eventually included for analysis. All demographic data are presented in Table 1. The median (range) ages were 69 years (42 years) in the NAION group and 68 years (37 years) in the NC group, and there were 63% male subjects in both groups. There were significant differences in the prevalence of systemic hypertension (*P* = 0.003) and self-reported OSAS (*P* = 0.003), but not for diabetes (*P* = 0.195), ischemic heart disease (*P* = 0.09), or dyslipidemia (*P* = 0.888). The percentage of eyes with a small cup-to-disc ratio was significantly higher in the NAION group than in the control group (73% vs. 13%, *P* < 0.0001).

**Table 1.** Demographic and Ophthalmic Characteristics of the Subjects

	NAION (*n* = 22)	Control (*n* = 30)	*P* Value
Demographic characteristics			
Age, years	69 (42)	68 (37)	0.213
Sex, M/F	14/8	19/11	0.982
Diabetes, %	36	17	0.195
Self-reported history of OSA, %	46	7	0.003^*^
Ischemic heart disease, %	23	7	0.09
Systemic hypertension, %	68	27	0.003^*^
Dyslipidemia, %	32	30	0.888
Ophthalmic characteristics			
IOP, mm Hg	16 (13)	15 (14)	0.644
Cup/disc < 0.3, number (%)	73	13	<0.0001^*^

Data are given as median (range).

NAION, nonarteritic anterior ischemic optic neuropathy; NA, not appropriate; OSAS, obstructive sleep apnea syndrome; IOP, intraocular pressure.

* Statistically significant result.

### Ophthalmic and OCT Data

**Figure 1.**
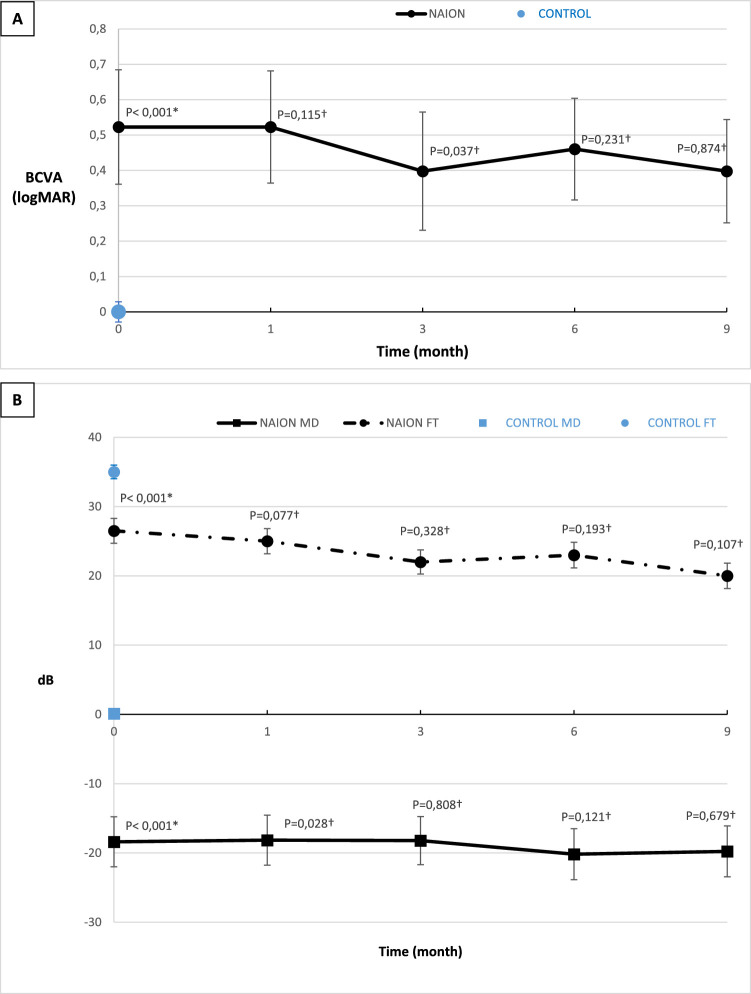
(**A**) Progression of median (standard deviation error bars) best-corrected visual acuity (BCVA) (logarithm of the minimum angle of resolution [log MAR] scale). (**B**) Progression of visual field parameters: median (standard deviation error bars) mean deviation (MD) and foveal threshold (FT). *Mann-Whitney bilateral test between the NC group value and the M0 NAION group value was performed. †Wilcoxon signed rank test with the precedent value was performed.

**Figure 2.**
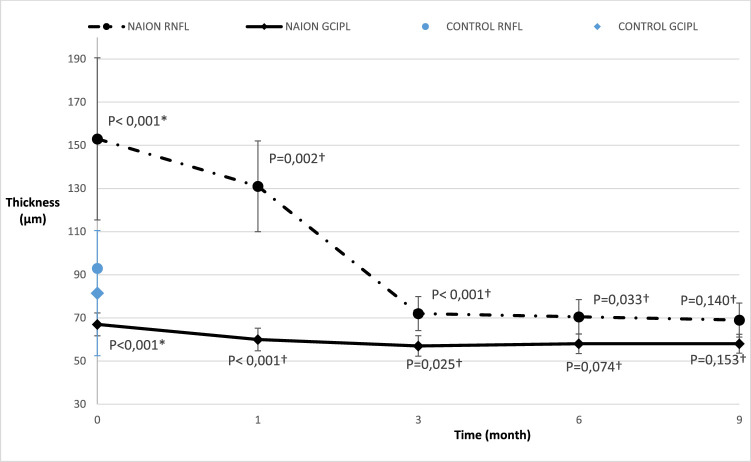
Progression of median (standard deviation error bars) peripapillary retinal nerve fiber layer (RNFL) and macular ganglion cell inner plexiform layer (GCIPL) thicknesses. *Mann-Whitney bilateral test between the NC group value and the M0 NAION group value was performed. †Wilcoxon signed rank test with the precedent value was performed.

Follow-up data are presented in Figures 1 and 2. In the NAION group, initial BCVA was 0.52 (1.26) log MAR in the acute phase (*P* < 0.0001 compared to the control group) and 0.40 (0.95) log MAR at M9. There was no significant change in BCVA between the acute phase and the final evaluation at M9 (*P* = 0.101) (see Fig. 1). Regarding visual field tests, mean deviation (MD) and foveal threshold (FT) were significantly decreased in the NAION group compared to controls (*P* < 0.0001) during the acute phase. However, there were no significant changes in MD and FT during follow-up (see Fig. 1). The median MD was -18.39 (24.35) dB at the acute phase and -19.77 (28.60) dB at M9, with no significant difference (*P* = 0.874). The FT was 26.5 (29) db in the acute phase, and 20 (24) at M9 (*P* = 0.019).

On spectral-domain OCT (SD-OCT), the peripapillary RNFL thickness was 93.0 µm (26 µm) in the NC group and 153.0 µm (285 µm) in the NAION group in the acute phase (*P* < 0.0001). The RNFL thickness decreased significantly at M1 (*P* = 0.002 compared to M0) and M3 (*P* < 0.0001 compared to M1) and remained stable at M6 and M9 (see Fig. 2). The GCIPL thickness was 81.5 µm (17 µm) in the NC group and 66.5 µm (38 µm) in the NAION group during the acute phase (*P* < 0.0001). The GCIPL thickness was 60.0 µm (43 µm) at M1, representing a significant decrease from the acute phase (*P* < 0.0001). The GCIPL thickness did not change significantly after M1 (see Fig. 2).

### OCT-A Data

**Figure 3.**
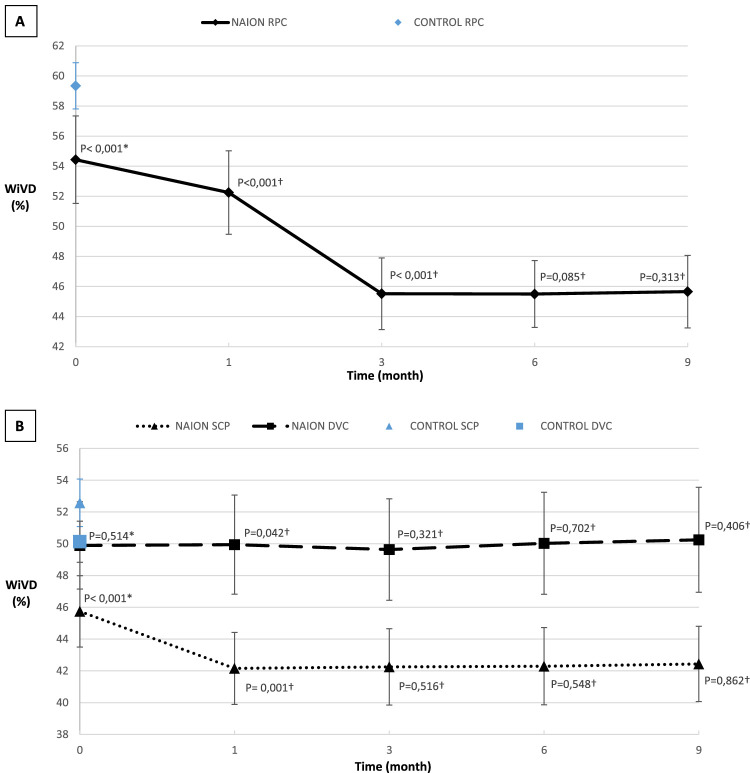
(**A**) Progression of median (standard deviation error bars) whole en face image vessel density (WiVD) in the retinal peripapillary capillary (RPC) plexus. (**B**) Progression of median (standard deviation error bars) WiVD in the superficial capillary plexus (SCP) and deep vascular complex (DVC). *Mann-Whitney bilateral test between the NC group value and the M0 NAION group value was performed. †Wilcoxon signed rank test with the precedent value was performed.

In the peripapillary region, the RPC WiVD was significantly decreased in the acute phase in the NAION group compared to the NC group: 54.43% (24.55%) and 59.35% (9.91%), *P* < 0.0001, respectively. During follow-up, the RPC WiVD was significantly decreased to 52.25% (26.11%) at M1 (*P* < 0.001), and to 45.52% (21.45%) at M3 (*P* < 0.0001 compared to M1). However, there was no further decrease: 45.50% (22.11%) at M6 (*P* = 0.085 compared to M3) and 45.66% (23.64%) at M9 (*P* = 0.313 compared to M6; Fig. 3).

Within the macula, the WiVD of the SCP was significantly reduced to 45.75% (17.6%) in the acute phase in the NAION group compared to 52.58% (10.38%) in the NC group (*P* < 0.0001). Over the course of follow-up, a significant decrease was observed only at M1: 42.16% (15.62%, *P* < 0.0001). SCP WiVD was 42.25% (17.13%) at M3, 42.30% (16.18%) at M6, and 42.44% (16.24%) at M9. At M3, M6, and M9, no change in WiVD SCP was observed in the NAION group compared to each previous visit (*P* = 0.516, *P* = 0.548, and *P* = 0.862, respectively; see Fig. 3). In the DVC, the WiVD was 49.90% (19.83%) in the acute phase in the NAION group and 50.13% (12.41%) in the NC group with no significant difference (*P* = 0.514). Over the course of follow-up, DVC WiVD was 49.95% (21.30%) at M1, DCP WiVD was 49.64% (21.16%) at M3, 50.03% (22.55%) at M6, and 50.25% (25.89%) at M9. At M1, M3, M6, and M9, no change in DCP WiVD was observed compared to each previous visit (*P* = 0.042, *P* = 0.321, *P* = 0.702, and *P* =0.406, respectively; see Fig. 3).

### Correlations

The overall correlations among visual parameters, OCT measurements, and OCT-A data are listed in Table 2 for the acute phase (M0) and in Table 3 for the atrophic phase (M9).

**Table 2.** Acute Edematous Stage: Spearman Coefficient Correlation Matrix for OCT-A, OCT, and Visual Parameters

		RPC WiVD	SCP WiVD	DVC WiVD
BCVA log MAR	*R*	−0.441	−0.208	−0.313
	*P*	0.041	0.350	0.156
MD	*R*	0.450	0.273	0.411
	*P*	0.037	0.217	0.059
FT	*R*	0.529	−0.148	0.453
	*P*	0.013	0.511	0.036
RNFL thickness	*R*	0.423	0.451	0.344
	*P*	0.051	0.037	0.118
GCIPL thickness	*R*	**0.591** ^*^	**0.604** ^*^	0.462
	*P*	**0.004** ^*^	**0.003** ^*^	0.032

BCVA, best corrected visual acuity; log MAR, log of the minimum angle of resolution; MD, mean deviation; FT, foveal threshold; RNFL, peripapillary retinal nerve fiber layer; GCIPL, macular ganglion cell-inner plexiform layer; RPC WiVD, radial peripapillary capillary whole en face image vessel density; SCP WiVD, superficial capillary plexus whole en face image vessel density; DVC WiVD, deep vascular complex whole en face image vessel density.

* Statistically significant correlation.

**Table 3.** Chronic Atrophic Stage: Spearman Coefficient Correlation Matrix for OCT-A, OCT, and Visual Parameters

		RPC WiVD	SCP WiVD	DVC WiVD
BCVA log MAR	*R*	**−0.562** ^*^	**−**0.266	**−**0.174
	*P*	**0.007** ^*^	0.231	0.438
MD	*R*	**0.518** ^*^	0.494	0.366
	*P*	**0.01** ^*^	0.021	0.094
FT	*R*	**0.593** ^*^	0.417	0.457
	*P*	**0.004** ^*^	0.054	0.034
RNFL thickness	*R*	**0.655** ^*^	0.393	0.104
	*P*	**0.001** ^*^	0.071	0.644
GCIPL thickness	*R*	0.195	**0.551** ^*^	0.407
	*P*	0.382	**0.009** ^*^	0.061

BCVA, best corrected visual acuity; log MAR, log of the minimum angle of resolution; MD, mean deviation; FT, foveal threshold; RNFL, peripapillary retinal nerve fiber layers; GCIPL, macular ganglion cell-inner plexiform layer; RPC WiVD, radial peripapillary capillary whole en face image vessel density; SCP WiVD, superficial capillary plexus whole en face image vessel density; DVC WiVD, Deep vascular complex whole en face image vessel density.

* Statistically significant correlation.

In the acute edematous phase (M0), there was no significant correlation between BCVA and RPC WiVD (R = −0.441, *P* = 0.041), SCP WiVD (R = −0.208, *P* = 0.350), or DVC WiVD (R = −0.313, *P* = 0.156). Similarly, there were no significant correlations between the visual field parameters (MD and FT) and the WiVD RPC (*P* = 0.037 and *P* = 0.013, respectively), the WiVD SCP (*P* = 0.217 and *P* = 0.511, respectively), or the WiVD DVC (*P* = 0.059 and *P* = 0.036, respectively). Similarly, there was no correlation between the RNFL thickness and the RPC WiVD (R = 0.423, *P* = 0.051), the SCP WiVD (R = 0.451, *P* = 0.037), or the DVC WiVD (R = 0.344, *P* = 0.118). However, there was a significant correlation between GCIPL thickness and RPC WiVD (R = 0.591, *P* = 0.004), as well as SCP WiVD (R = 0.604, *P* = 0.003), but not with DVC WiVD (R = 0.462, *P* = 0.032; see Table 2).

In the chronic phase (M9), there was a significant correlation between RPC WiVD and BCVA (R = −0.562, *P* = 0.007), as well as with visual field parameters: MD (R = 0.518, *P* = 0.01) and FT (R = 0.593, *P* = 0.004). There was also a good correlation between RPC WiVD and RNFL thickness (R = 0.655, *P* < 0.001), but not with GCIPL (R = 0.195, *P* = 0.382). SCP WiVD was not correlated with BCVA (R = −0.266, *P* = 0.231), MD (R = 0.494, *P* = 0.021), FT (R = 0.417, *P* = 0.054), or RNFL thickness (R = 0.393, *P* = 0.071). However, the SCP WiVD was correlated with the GCIPL (R = 0.551, *P* = 0.009). DVC WiVD was not correlated with BCVA (R = −0.174, *P* = 0.438), MD (R = 0.366, *P* = 0.094), FT (R = 0.457, *P* = 0.034), RNFL thickness (R = 0.104, *P* = 0.644), or GCIPL (R = 0.407, *P* = 0.061).

## Discussion

To our knowledge, this study is the first to prospectively analyze OCT-A data from a relatively large cohort of patients with NAION over a 9-month period of time. Compared to our previous retrospective study,^5^ we report 4 new results: (1) the rarefaction of RPC observed on OCT-A began in the acute edematous phase and became more pronounced during progression to the chronic atrophic phase; (2) the macular SCP capillaries are affected early but, unlike the RPC, the decrease in VD ceased to progress after 1 month; (3) the decrease in macular VD in the SCP was correlated with anatomic involvement of the retinal ganglion cell axons in both the acute and chronic phases; and (4) the macular DVC was not affected during all the follow-up visits.

**Figure 4.**
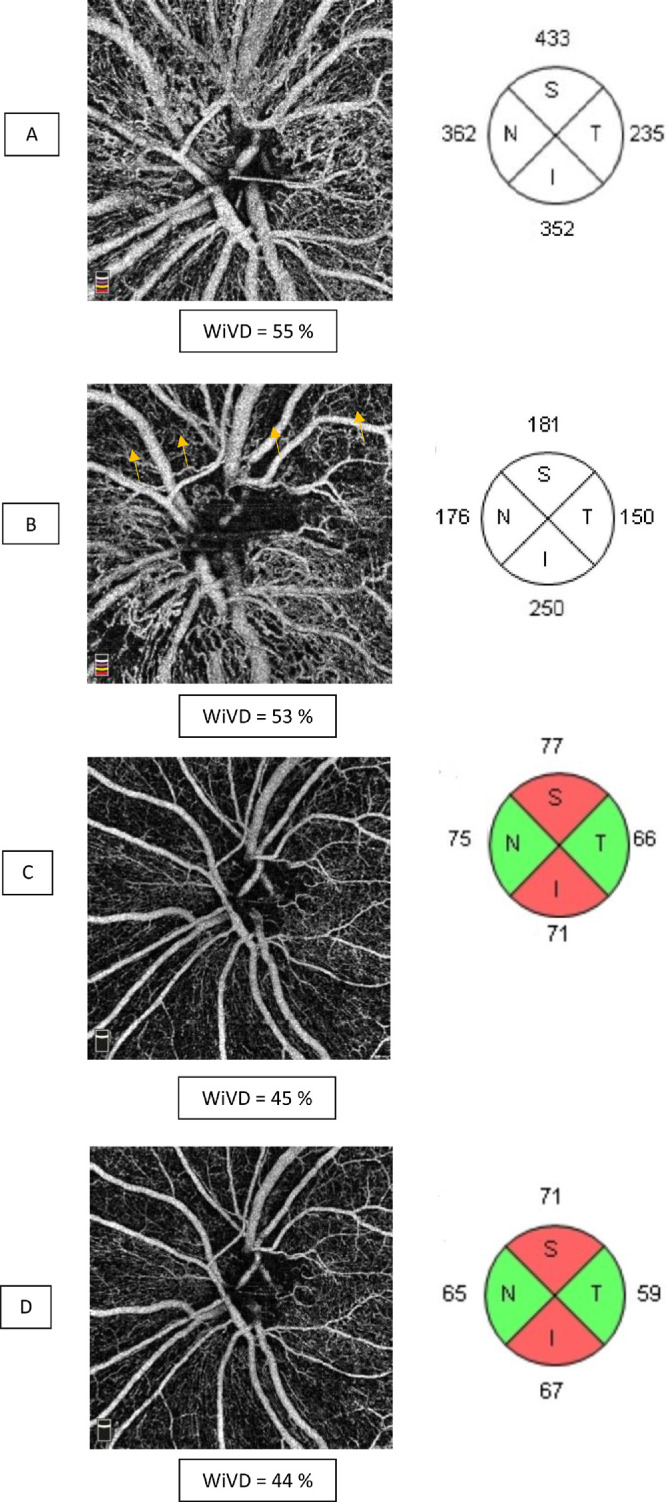
Six-month follow-up of a case of NAION. Left column: OCT-A en face images segmented in the radial peripapillary capillary plexus (RPC). Right column: OCT retinal nerve fiber layer (RNFL) thickness average in the four quadrants. (**A**) At diagnosis, there is disc edema with a significant increase in RNFL thickness; however, the vessel density in the RPC is only slightly decreased. (**B**) One month later: decreased disc edema, particularly in the upper quadrant, where capillary areas are thin appears (*arrows*). (**C**) At 3 months follow-up, the disc edema is resorbed, and there is an atrophic optic disc with a thinned RNFL (mainly in the upper and lower quadrants). There is prominent capillary rarefaction in these areas. (**D**) At 6 months follow-up, there is no major change.

RPC impairment in the acute phase of NAION, which was previously only suspected with fluorescein angiography, was thus demonstrated using OCT-A. As in other recent studies,^18^^,^^19^^,^^22^ we found a decrease in VD values in the RPC in the acute phase of NAION (see Fig. 3). Although some studies have reported cases showing an improvement in RPC VD during resorption of the edema, we observed a worsening in capillary depletion at the 1- and 3-month visits (see Figs. 3, 4). The mechanism explaining this peripapillary capillary rarefaction remains poorly understood. Indeed, unlike arteritic ischemic optic neuropathy^23^ or central retinal artery occlusion,^24^ which are complete vascular occlusions generating downstream capillary ischemia by stopping the perfusion, NAION results from a merely transient hypoperfusion of the ONH. This low flow is related to circulatory slowing in the PCAs,^3^ often due to prolonged nocturnal hypotension^2^ in OSAS^3^ (see Table 1). Consequently, the decrease in RPC WiVD measured in the acute phase (see Fig. 3) cannot only result from hypoperfusion of the PCAs, as this is only a transient phenomenon and is typically no longer present by the time of diagnosis and OCT-A evaluation. It has been suspected that this decrease in RPC density could be related to disc edema through a compressive mechanism.^5^^,^^17^ RPCs interconnect within the RNFL,^25^ where they play a supportive metabolic role. Compression of the RPC within the edematous RNFL might lead to circulatory slowing within the capillaries, which would be interpreted as a decrease in VD on OCT-A. However, in a study comparing acute NAION with papilledema, Fard et al.^26^ found decreased VD levels in the NAION group unrelated to the extent of disc edema as measured by RNFL thickening on SD-OCT. This important result demonstrated that disc edema alone was not responsible for the reduction in RPC WiVD. In addition, if axonal swelling resulted in decreased blood flow by compression of the capillaries, resolution of the disc edema in NAION (around the third month following the acute episode^27^) should improve capillary perfusion. However, our results showed the opposite, with a worsening of VD over the course of follow-up after the acute phase of NAION (see Fig. 4).

**Figure 5.**
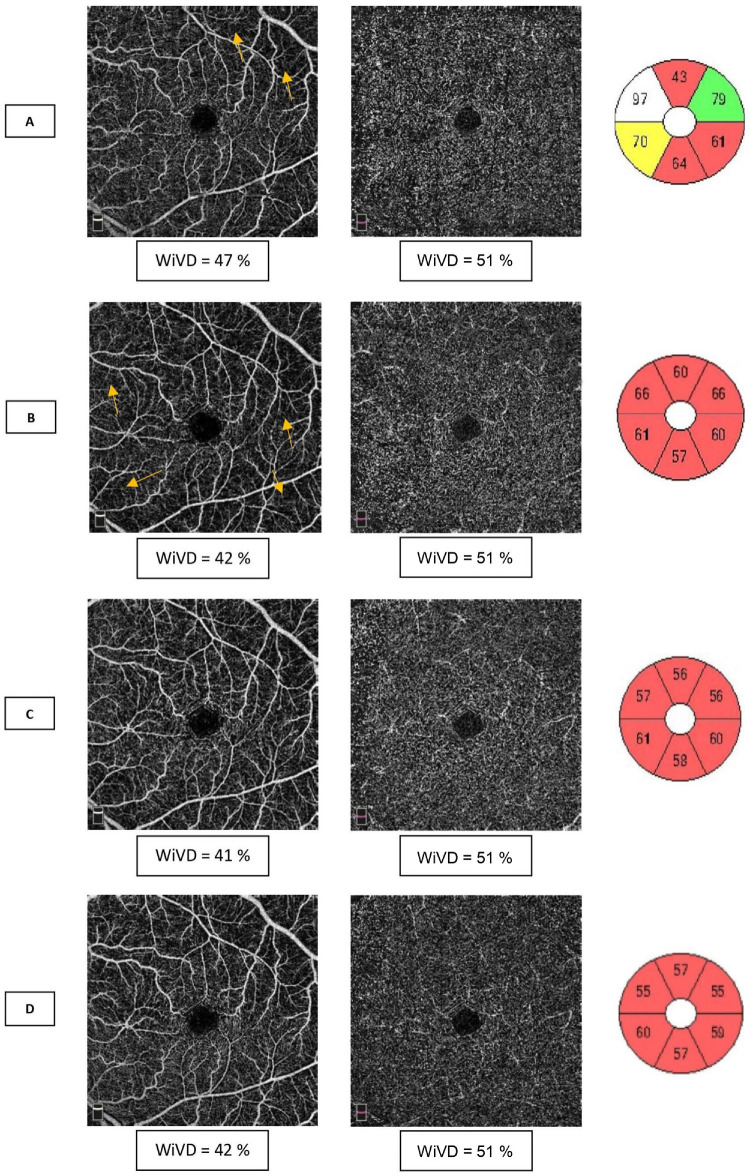
Same patient as Figure 3. Macular OCT-A images of the superficial capillary plexus (SCP) (*first column*) and deep vascular complex (DVC) (*second column*) with average OCT ganglion cell and inner plexiform layer (GCIPL) thickness (*third column*) in the six quadrants. (**A**) At the time of diagnosis, there is a decrease in whole en face image vessel density (WiVD) in the SCP with localized areas of capillary rarefaction (*arrows*). The DVC is not affected. There is edema of the nasal GCIPL, but thinning of the temporal quadrants is already present. (**B**) One month later, there is a capillary depletion in the SCP associated with thinning of the GCIPL in all quadrants. At 3 (**C**) and 6 (**D**) months follow-up, there is no significant change.

It is more likely that in NAION, the decrease in VD is a response to the loss of the active metabolic tissue, the RNFL, as mentioned by Rebolleda et al.^19^ and found in other optic neuropathies, such as glaucoma.^9^^,^^11^^,^^28^^,^^29^ This phenomenon is similar to what has been observed in the macular area (Fig. 5). We found a decrease in macular VD in the SCP starting in the acute phase. However, in contrast to RPC, which progressively decreased during the months of resorption of the disc edema, macular SCP damage was significant in the acute phase and stabilized after only 1 month (see Fig. 3). In fact, we found no significant differences in VD between M1 and M9 in the NAION group in any of these capillary plexuses. Concerning the DVC, we found no difference between the control group and the NAION group in either the acute or atrophic phase. These results are in agreement with the literature, and show that unlike other optic neuropathies, such as glaucoma, NOIAA specifically affects the superficial capillary plexuses.^7^

These kinetics of damage to macular structures might be related to the lesions in ganglion cells, which appear altered early on SD-OCT,^30^ largely before resolution of the RNFL edema.^31^ The timing of ganglion cell atrophy differs between studies.^19^^,^^32^ This apparently discordant result can be explained by the use of ganglion cell complex or GCIPL as a measurement method.^17^ The GCIPL does not include the RNFL,^33^ which can be edematous for 6 months,^34^ thus an earlier atrophy of the GCIPL was observed ∼ 1 month after the onset of symptoms.^27^ In the present study, we also observed thinning of the GCIPL in the NAION group in the acute phase, with worsening at M1. No significant differences between M1 and the later evaluations in the chronic phase were found, similarly to the capillary measurements. In addition, the GCIPL thinning was concomitant (see Fig. 2) and correlated (see Tables 2, 3) with the decrease in SCP WiVD. These results may confirm that the atrophy of the retinal nerve tissue (mainly RGCs and their axons) might lead to a lower metabolic demand, resulting in decreased capillary perfusion. This decrease in flow is observed as a decrease in VD on OCT-A images^12^ (see Fig. 4). So far, information on whether or not capillaries are lost has remained limited due to the detection threshold of OCT-A. Nevertheless, it seems that the mechanism of blood flow autoregulation continues despite the atrophy, avoiding over-oxygenation of the retina, which would accelerate neurodegeneration through production of reactive oxygen species. In all recent publications, it is clear that whatever the cause of the degeneration of RGCs, including central neurological diseases,^35^^,^^36^ there is an associated thinning of the superficial capillary plexus. In glaucoma, the RPCs are altered, because they vascularize the RNFL, ganglion cell layer, and IPL, which are the most affected tissues in this pathology.^37^ These new in vivo OCT-A data confirm earlier results, which showed that, as in the brain, blood flow in the ONH^38^ and retina^39^ adapts to neuronal activity.^40^

In progressive pathologies, the quantification of macular perfusion thus makes it possible to detect dysfunction of RGCs before they enter into a process of apoptosis and cause thinning of the GCIPL seen on SD-OCT. This may explain why different VD values have been found between groups of patients with atrophic NAION and patients with glaucoma despite similar RNFL and GCIPL thicknesses.^7^

Our analysis is not free of bias, particularly regarding measurements of DVC, which is most often overestimated due to projection artifacts. Nevertheless, this has recently been improved by the emergence of projection artifact removing algorithm^20^^,^^21^ (see Fig. 4). In addition, this bias is the same in the control and NAION groups, as well as during monitoring, which limits its statistical impact. Moreover, the presence of edema in the retinal layers is a phenomenon that may have altered the quality of the signal, which underestimates acute phase capillary density measurements. The evolution of the edema may therefore have affected the comparability of OCT-A results over time in the NAION group. Nevertheless, this bias does not change the interpretation of the results. Indeed, it was a reduction of the RCP and SCP WiVD that was observed during the follow-up. The 9-month follow-up period might be too short, but NAION studies usually consider OCT and visual parameters stable even before this time^34^ (usually 6 months).

In conclusion, this longitudinal study showed that in patients with NAION, VD analysis of the capillary plexus provides rapid and objective data on the state of the RGCs and their axons, unlike SD-OCT measurements, which remain overestimated due to edema. The NAION OCT-A study provides an in vivo analysis of the relationship between atrophy of the retinal nervous and vascular tissues, which may improve our understanding of other slowly progressive neuro-ophthalmological pathologies.
